# Comparing Intravenous Labetalol and Intravenous Hydralazine for Managing Severe Gestational Hypertension

**DOI:** 10.7759/cureus.42332

**Published:** 2023-07-23

**Authors:** Eustace Ehikioya, Okelue E Okobi, Maame Akosua E Beeko, Rafia Abanga, Namtor Nicole I Abah, Lilian Briggs, Patience N Nwimo, Papa Kwame A Beeko, Onyinyechukwu B Nwachukwu, Caroline C Okoroafor

**Affiliations:** 1 Obstetrics and Gynecology, Edo Specialist Hospital, Benin, NGA; 2 Family Medicine, Medficient Health Systems, Laurel, USA; 3 Family Medicine, Lakeside Medical Center, Belle Glade, USA; 4 Internal Medicine, Kyebi Government Hospital, Kyebi, GHA; 5 Obstetrics and Gynecology, Weija Gbawe Municipal Hospital, Accra, GHA; 6 Neuroscience, Mater Dei Hospital, Msida, MLT; 7 Internal Medicine, Grodno State Medical University, Belarus, AUS; 8 Internal Medicine, First Foundation Medical Clinic, Loganville, USA; 9 General Internal Medicine, Suhum Government Hospital, Suhum, GHA; 10 Internal Medicine, American International School of Medicine Georgetown, Guyana, USA; 11 General Practice, University of Calabar, Calabar, NGA

**Keywords:** pregnancy, gestational hypertension, severe hypertension, intravenous labetalol, intravenous hydralazine

## Abstract

Background

Hypertensive disorders of pregnancy are the leading causes of both maternal morbidity and maternal mortality. Hypertensive disorders are acute obstetric emergencies, which refer to various life-threatening medical challenges known to develop during pregnancy, labor, and delivery, requiring urgent attention to reduce blood pressure (BP) for the benefit of the affected mothers and infants. Hydralazine and labetalol have been widely used as the first-line medications in the management of severe hypertension during pregnancy. However, the choice between these two drugs lacks clear evidence regarding their safety and superiority. Several studies have attempted to study intravenous (IV) labetalol versus hydralazine, but very few such comparison studies have been conducted in Africa.

Objective

To compare the effectiveness of IV labetalol and IV hydralazine in reducing systolic and diastolic BP in pregnant women with severe hypertension. Also, to determine the time required for hydralazine and labetalol to lower BP to ≤150/100 mmHg, the number of doses needed for each drug, and evaluating maternal and perinatal outcomes.

Study design

This study employed an open-label randomized clinical trial design conducted in the labor, delivery, and antenatal ward of the Central and Stella Obasanjo Hospital in Benin City. A total of 120 women with severe pregnancy-induced hypertension were randomly assigned to two groups: Group X, consisting of 60 pregnant women, received IV hydralazine at a slow rate of 5 mg for five minutes, repeated every 20 minutes (maximum of five doses) until a blood pressure of ≤150/100 mmHg was achieved. Group Y, also consisting of 60 pregnant women, received IV labetalol in escalating doses of 25, 50, 75, 75, and 75 mg (maximum of 300 mg) every 20 minutes until the blood pressure reached ≤150/100 mmHg. Statistical analysis was performed using SPSS version 23 (IBM Inc., Armonk, New York).

Result

IV hydralazine achieved the target BP in an average time of 45.80 +/- 25.17 minutes, while IV labetalol took an average of 72.67 +/- 41.80 minutes (p=0.001). The number of doses required to reach the target BP differed significantly between the two drugs. Hydralazine required an average of 1.72 +/- 0.904 doses, whereas labetalol required an average of 3.72 +/- 1.782 doses (p=0.0001). While 45% of women in the hydralazine group attained the target BP with a single dose of hydralazine, only 31.1% of women in the labetalol group were able to attain the target BP with a single dose of labetalol (p=0.02). Overall, target BP was achieved in 55 out of 60 women (91.7%) who were randomized to receive IV hydralazine, whereas 45 out of 60 women (75%) who received IV labetalol achieved the target blood pressure. While hydralazine demonstrated more favorable results in terms of achieving target blood pressure, there were higher incidences of maternal adverse effects in the hydralazine group compared to the labetalol group. However, these adverse effects were not severe enough to warrant discontinuation of the medication.

Conclusion

IV hydralazine showed faster achievement of the target BP and a lower number of doses required compared to IV labetalol. Additionally, a higher percentage of women in the hydralazine group achieved the target BP with a single dose. However, there were more maternal adverse effects associated with hydralazine, although they were not severe. Perinatal outcomes did not differ significantly between the two groups.

## Introduction

Gestational hypertension is a medical disorder in pregnancy marked by high blood pressure (BP) levels that develop during pregnancy, specifically after ≥20 weeks of gestation, in the absence of organ dysfunction, signs of preeclampsia, and proteinuria. After parturition, the BP typically returns to normal [1]. Gestational hypertension can be categorized into three levels of severity. Mild gestational hypertension has been described as a systolic BP of 140-149mmHg, or diastolic BP of 90-99 mmHg, even as severe gestational hypertension has been defined as a systolic BP of 160 mmHg and higher, or diastolic BP of 110 mmHg and higher [2, 3]. Gestational hypertension is a significant cause of both maternal mortality and maternal morbidity, particularly in sub-Saharan Africa [2]. Throughout the globe, nearly 5-8% of pregnancy complications are attributable to hypertension [2, 3]. One in every ten women tend to develop severe gestational hypertension during their initial pregnancy, which often leads to complications like liver, kidney, respiratory problems, and cerebral hemorrhage. It is estimated that over 63,000 women lose their lives each year due to hypertension-related complications, with more than 90% of these deaths occurring in developing countries [4]. Nevertheless, it is worth observing that women diagnosed with gestational hypertension are also likely to develop preeclampsia, which is diagnosed when a pregnant woman has increased quantities of protein in the urine. In the United Kingdom, preeclampsia ranks as the second leading cause of direct maternal death and perinatal mortality. Thus, in the US, preeclampsia is responsible for approximately six to nine deaths in pregnant women and over 175 babies annually [5-7]. In particular, eclampsia is regarded as a major cause of maternal deaths, as it accounts for approximately 3% of deaths occurring during deliveries in Central Hospital, Benin City. In comparison to other hospitals and centers in Nigeria, the 3% eclampsia-attributed mortality rate for women during delivery is higher than in Jido, Kano with 1.2% death rates, Onuh with 1.32% death rates, and other centers in the country with a combined death rate of 0.82% [6-9].

Developing effective strategies for the prevention and treatment of gestational hypertension has been challenging due to the elusive understanding of its underlying mechanisms. Hypertension in a normal pregnancy follows a dynamic pattern: BP decreases during the first trimester are attributed to pregnancy hormones, including estrogen, relaxin, and progesterone, which dilate the vascular smooth muscles of the endothelium [9]. Subsequently, BP gradually rises in the second trimester and returns to its pre-pregnancy state by the end of pregnancy. However, individuals at risk of hypertensive disorders in pregnancy do not experience this normal pattern, leading to a sustained increase in BP.

Hypertensive disorders in pregnancy can be categorized as follows: (a) gestational hypertension, occurring in previously normotensive women after 20 weeks of gestation, without significant proteinuria; (b) severe hypertension, defined by a systolic BP of ≥160 mmHg or diastolic BP of ≥110 mmHg, and mainly diagnosed in previously hypertensive women or when hypertension occurs prior to 20 weeks of the gestation period, without molar pregnancy and connective tissue disease; (c) preeclampsia is the pregnancy-specific disorder that is marked by the extensive vasospasm and endothelial dysfunction that normally occur after 20 weeks of gestation, in addition to presenting as late as four weeks postpartum; and (e) eclampsia, which involves convulsions along with hypertension [7]. Additionally, chronic hypertension, which is mainly diagnosed as per the American College of Obstetricians and Gynecologists (ACOG) guidelines, as in-office measurement, and entails a systolic BP higher than 140mmHg or a diastolic BP higher than 90mmHg established through ambulatory BP monitoring, home BP monitoring, and BP assessment with successive office visits, and with higher pressures at least four hours apart before 20 weeks gestation, may be superimposed on preeclampsia. Maternal complications of preeclampsia may include convulsions, cerebrovascular accidents, myocardial infarction, renal failure, disseminated intravascular coagulopathy, hemolysis elevated liver enzymes and low platelets (HELLP) syndrome, abruptio placentae, as well as fetal complications like intrauterine growth restriction, fetal distress, and fetal death [2, 3, 8].

To prevent these complications, rapid-acting antihypertensive drugs are often administered with the objective of reducing and controlling the BP without having any adverse effects such as hypotension and fetal distress on the fetus and mother. Once BP is adequately controlled, the decision to deliver the baby depends on gestational age and the presence or absence of complications. Near-term or term pregnancies are typically delivered immediately through the fastest and safest route. However, in cases of extreme prematurity without complications but with well-managed BP, conservative management may be considered in selected centers to improve fetal outcomes [10].

Antihypertensive pharmacology during pregnancy

There are several classes of antihypertensive medications available for reducing BP. Currently, while several studies and clinical trials have been conducted in Nigeria in relation to the choice of antihypertensive drugs for managing severe hypertension in pregnant women, many clinicians still rely on the findings of studies conducted in Europe for guidance. Hydralazine, nifedipine, and labetalol have been suggested as the first-line medication for a quick reduction of BP. However, there is no consensus on which of these drugs is superior in this specific setting [11]. At Central Hospital Benin, the primary site of this study, IV labetalol has been used as the drug of choice empirically since its introduction in 2011. Prior to that, IV hydralazine was utilized. This study aims to provide evidence-based clinical data to assist in selecting the appropriate antihypertensive agent for managing severe hypertension during pregnancy at Central Hospital Benin City, Nigeria.

Physiological changes associated with hypertension in pregnancy and choice of antihypertensive

The physiological changes that occur during pregnancy can have significant effects on the pharmacokinetics of drugs, including absorption, distribution, metabolism, protein binding, and excretion. Pregnancy is often marked by an increment in the heart rate (HR) alongside stroke volume (SV), which causes general increments in cardiac outputs (CO) (CO = HR x SV). However, despite this increase, arterial BP decreases due to a reduction in total peripheral vascular resistance in the mid-trimester [9].

The hemodilution and volume expansion in pregnancy result in a progressive decrease in albumin concentration, which in turn reduces the plasma protein binding of certain antihypertensive medications that are albumin binding. Additionally, the increment in cardiac output during pregnancy leads to the increment of the effectual renal plasma flow (about 60-70%), the rate of glomerular filtration (about 50%), and clearance of creatinine, leading to increased clearance of drugs eliminated through the kidneys [10, 11]. To this end, Lobstein et al. disclosed that the maternal hepatic clearance, improved through the cardiac output increment, was affected by the drug-metabolizing enzyme changes that occur during pregnancy, attributable to the pregnancy hormones' effects on the cytochrome P450 enzymes [11].

Considering the metabolic changes during pregnancy is crucial when prescribing rapid-acting antihypertensive drugs and determining the appropriate dosage for treatment. The dosing frequency may need to be increased for drugs with shorter durations of action. The beta-adrenoceptor blockers medications' therapeutic effects may be assessed by monitoring the drug's serum levels and indirectly by observing the patient's heart rate. By understanding the fundamental pharmacology of drugs in pregnancy and considering clinical observations of adverse effects, healthcare providers can make informed adjustments to the dose and dosing interval to achieve the desired therapeutic effect. A thorough comprehension of the physiological changes during pregnancy and the gestation-specific pharmacology of different antihypertensives is essential for effective treatment and minimizing risks to both the mother and the fetus. For instance, in the management of severe pregnancy-related hypertension, avoiding hypotension is vital, given that the degree of regulation of placental blood flow has not been aptly established, and excessively aggressive reduction of BP might lead to fetal distress [12-21].

The adrenergic receptor antagonist labetalol peripheral acting

Extensive utilization of beta-blockers occurs during pregnancy. Despite the observation that numerous randomized trials have compared beta-blockers to other agents and placebo [22-29], a number of issues regarding the use of the drug during pregnancy remain unresolved. These concerns primarily arise from a limited number of small studies suggesting a potential association between beta-blockers and infants born with low birth weights. Nevertheless, of the existing beta-blockers, none has been associated with teratogenicity. A recent Cochrane review and meta-analysis [30] found that it was challenging to distinguish the perinatal effects of individual beta-blockers, except for atenolol. One small study observed that when atenolol was initiated between 12 to 24 weeks of gestation, it significantly increased the risk of restriction of intrauterine growth alongside the risk of reduction in placental weight in comparison to placebo [31]. Even as beta-blockers vary with regard to factors such as receptor specificity and lipid solubility, no extensive evaluation has been conducted on their possible clinically pertinent divergences during pregnancy. Still, non-clinically significant neonatal bradycardia has been linked to the use of oral beta-blockers [32, 33]. Nevertheless, a recent systematic review of various randomized controlled trials disclosed that labetalol did not appear to have an impact on neonatal heart rate [34]. In contrast, a study found that parenteral administration of beta-blockers increased the neonatal bradycardia risk, which requires urgent intervention in every one of six newborns [32]. Additional reassurance has been drawn from the one-year postpartum follow-up research, which showed typical development in babies/infants who had been exposed to atenolol while in the uterus [35]. Improvements in maternal outcomes have been observed in instances of beta-blockers use, given their ability to efficiently control and manage maternal BP, lower the severe hypertension incidence, and lower the preterm hospitalization rate. One of the most recent Cochrane analyses disclosed that beta-blockers were increasingly effective in comparison to methyldopa in reducing BP [32-35].

Labetalol is an adrenergic receptor antagonist that exhibits both non-selective and selective beta-adrenergic receptor-blocking action. Its beta-blockade/alpha-blockade ratio is seven to one [35-37]. Stimulation of alpha-adrenergic receptors leads to vasoconstriction of arteriolar and venular smooth muscles, resulting in increased peripheral resistance. On the other hand, activation of beta-adrenergic receptors increases cardiac output by raising heart rate, conduction velocity, stroke volume, and the rate of myocardial relaxation. The effect is mainly realized through the increment of the calcium ions sequestration rate, which is a positive lusitropic effect, and, as a result, increases heart rate. Moreover, labetalol hydrochloride is considered a racemic mixture, which is also chemically designated as 5-(1-hydroxy-2-((1-methyl-3-phenylpropyl)) amino ethyl) salicylamide monohydrochloride[1, 35-37]. 

Labetalol is considered safe during pregnancy as it crosses the placenta in small amounts and does not seem to present any teratogenic side effects on the basis of a significant body of evidence [2, 37]. The maximum dose of parenteral labetalol is 300 mg, administered incrementally in doses of 25mg, 50mg, 75mg, 75mg, and 75mg every 20 minutes until the desired BP is achieved. Labetalol is increasingly being recognized as the first-line drug of choice for managing severe hypertensive disorders during pregnancy due to its safety profile compared to other beta-blockers [3, 37]. However, a study by Peterson et al. disclosed a high prevalence rate of small-for-gestational-age (SGA) infants in certain patients who underwent labetalol treatment [38]. Peterson and colleagues also reported that labetalol's risk profile in pregnancy is not significantly different from that of other beta-blockers, as two studies indicated similar outcomes for pregnancies exposed to labetalol and other beta-blockers [38, 39]. Consequently, further comprehensive studies are required to validate the effect of labetalol on BP control, as well as its impact on maternal and neonatal outcomes, while also comparing it with other antihypertensive agents used for acute BP control. This need arises from the increasing use of labetalol and the uncertainties surrounding its effects and potential side effects during pregnancy.

Direct vasodilators (hydralazine)

Direct vasodilators, specifically hydralazine, are potent antihypertensive drugs known for their rapid action. Hydralazine, a hydrazine derivative, selectively relaxes smooth muscles in arterioles. However, the precise mechanism of its action remains unknown [12, 37]. This medication is effective when taken intravenously, orally, and intramuscularly. Thus, IV hydralazine administration is principally vital for swiftly controlling hypertension. When taken orally, hydralazine is well absorbed but undergoes rapid metabolism in the liver during the first pass, resulting in a bioavailability of only 25%. Acetylation plays a role in its metabolism, which exhibits a bimodal distribution in the population [37]. Individuals classified as rapid acetylators experience faster first-pass metabolism, leading to lower bioavailability of hydralazine and a subsequent reduction in its antihypertensive effect compared to slow acetylators. Hydralazine's half-life is estimated to be two to four hours, yet its vascular effects endure longer than its blood concentration [10, 37]. The typical daily dosage for parenteral hydralazine is 40mg, while oral hydralazine is usually prescribed at 200mg per day [10, 37]. Due to the aforementioned reduced bioavailability, oral hydralazine requires administration two to three times daily to maintain smooth control of BP. Before the introduction of labetalol in Central Hospital Benin City, Nigeria, hydralazine served as the preferred drug for rapidly controlling severe hypertension since 2011.

The adverse effects of hydralazine primarily stem from excessive vasodilation and sympathetic stimulation. Immediate side effects may include palpitations, headaches, flushing, and nausea. Lengthy usage can result in pyridoxine-responsive polyneuropathy and rare immunological reactions, such as lupus syndrome that is drug-induced. Administration of hydralazine has occurred in every pregnancy trimester without evidence of teratogenicity, although there have been reports on cases of lupus syndrome and neonatal thrombocytopenia [40]. Even as it was extensively utilized in the treatment of acute hypertension in the second and third trimesters, the drug's use has, over time, been replaced by agents with increasingly desirable and tolerable adverse effects profiles [41]. Thus, in relation to acute hypertension during the final pregnancy phases, the use of IV hydralazine has been linked to an increment in perinatal and maternal side effects in comparison to the administration of IV labetalol and oral nifedipine [42], such as an increment in the rates of cesarean sections, maternal hypotension, placental abruption, oliguria, and poor appearance, pulse, grimace, activity, and respiration (APGAR) scores [32]. Additionally, the more pronounced hydralazine side effects, including nausea, vomiting, and headaches, often appear as symptoms of worsening preeclampsia. The impact of hydralazine on uteroplacental blood flow remains unclear, with variations in reflex sympathetic activation likely being the cause. Maternal hypotension resulting from hydralazine administration may lead to fetal distress [43-52]. A recent meta-analysis of several randomized control trials evaluating the IV hydralazine use in the control of severe hypertension during pregnancy disclosed that oral nifedipine and IV labetalol were considered ideal as first-line medications, while hydralazine was considered an appropriate second-line option [42]. This study aims to further validate or challenge these findings, excluding nifedipine from the trial.

Timing for IV hydralazine and labetalol to achieve 150/100 mmHg or below target BP

The American College of Obstetricians and Gynecologists [43] maintains that these medications are prescribed as first-line treatments in instances of severe hypertension during pregnancy. The use of these drugs doesn't necessitate cardiac monitoring or specialized tools [44]. Previous studies conducted on Caucasian populations reported that labetalol and hydralazine met the criteria as first-line antihypertensive, with BP control achieved within an average time of 40 to 60 minutes [45-49]. However, it should be noted that the effectiveness of beta blockers in controlling hypertension may differ among Black individuals [50-51]. Moreover, drug potency issues in tropical environments [52] require further investigation to determine the most effective treatment approach. In a study conducted in a Nigerian teaching hospital, Numbur et al. found that both hydralazine and labetalol required approximately 40 minutes to achieve BP control, devoid of any statistically significant divergence in the two groups (p=0.17) [53]. These findings align with previous studies by Vigil-De Gracia et al. [45] and Delgado De Pasquale et al. [54]. Vigil-De Gracia et al.'s study involving 200 women with severe pregnancy-induced hypertension concluded that both hydralazine and labetalol are effective antihypertensive drugs for treating severe hypertension in pregnancy, devoid of observable significant differences with regard to persistent severe hypertension and maternal hypotension between the two medications [45]. However, the study did not specifically assess the time required for every medication to realize the targeted BP level as an appropriate effectiveness measure. Another randomized study by Deka et al. [55] compared hydralazine and labetalol in relation to their efficiency in managing pregnancy-induced hypertension and found that both drugs achieved the target BP within a short period, with a mean time of 32 minutes for hydralazine and 31 minutes for labetalol, which was not statistically significant (p=0.401383). Nevertheless, another meta-analysis by Duley et al. [18] disclosed inadequate data to make dependable conclusions regarding the two antihypertensive drugs' comparative effects. Previous studies predominantly favored the null hypothesis, indicating no superiority of one drug over another in achieving BP reduction [45, 54-58]. A study by Ayesha and Sadiza suggested that IV labetalol considerably and effectively reduced the mean arterial BP (MAP) compared to hydralazine, but it did not assess the time taken for both drugs to reduce BP to ≤150/100 mmHg and BP readings were not maintained on the study proforma after two readings [56]. Mable et al. additionally disclosed that hydralazine was more effective in lowering MAP in comparison to labetalol (13.3 vs. 11.2 mmHg) [57]. The finding was additionally corroborated by the randomized controlled trial carried out in Port Harcourt, Nigeria, and disclosed that hydralazine use took only 28 minutes to enable the reduction of the mean arterial BP to targeted levels, in comparison to the 75 minutes that took to realize similar outcomes [52]. Currently, there is limited evidence from randomized clinical trials comparing IV hydralazine and labetalol use in the management of acute hypertensive emergencies during pregnancy. Thus, a systematic review of several studies comprising 2,949 female participants concluded that despite the two antihypertensive medications efficiently lowering BP, insufficient evidence makes it challenging to determine the most effective drug for pregnant women with hypertension, indicating the prerequisite for additional studies in the area [52]. The present study's objective entails the comparison of the time required to reach the therapeutic goal and the dosages required after using IV hydralazine and labetalol in severe pregnancy-induced hypertension.

The dosages required to achieve BP levels below or equal to 150/100 mmHg (IV labetalol and hydralazine)

Limited research has been conducted on the number of doses required for labetalol and hydralazine to achieve a BP level of 150/100mmHg or lower [45, 54, 58-61]. Some studies found that an average of three doses of labetalol and a single dose of hydralazine were needed to achieve BP control [52, 61-65]. However, this study was criticized for its small sample size and lack of generalizability. The authors recommended further multi-center studies to explore alternative options to hydralazine and suggested it as the preferred drug for severe hypertension during pregnancy. Another study by Numbur et al. disclosed that 77.7% of participants placed in the hydralazine group and 81% of participants placed in the labetalol group needed a total of three dosages of the corresponding medications to realize the desired BP control; however, the difference was statistically insignificant [53]. Conversely, Ayesha and Khan randomized 78 women to receive labetalol or hydralazine and found that a single dose of labetalol was sufficient to lower the mean arterial BP to targeted levels in 51.3% of the participants, in comparison to 35.9% of participants placed in hydralazine group. The mean number of doses required was not statistically significant (1.6 versus 1.9), although the study had a small sample size [59]. Deka et al. conducted a comparative study in India and reported that 48% of patients achieved target BP after a single dose of hydralazine, while 30% required two to three doses and nine patients needed four to five doses. In the labetalol group, 50% of patients required a single dose, with 28% and 16% requiring two to three and four to five doses, respectively. These results showed no substantial difference between hydralazine and labetalol with regard to the number of doses required for BP control [55]. Another study in Pakistan found that an average of 2.74 doses and a 41-minute period were sufficient to lower BP in women receiving hydralazine [59]. Further, a study by Puvi et al. disclosed that, of the female participants, 81.5% placed in the labetalol group along with 69.5% placed in the hydralazine group needed only a dose to realize the targeted BP level, implying the ability of IV labetalol to act faster compared to IV hydralazine [60]. Comparable findings were reported in the study by Shabnum Tariq in 2017, which revealed that, compared to hydralazine, labetalol was highly effective [61]. Pooja et al. also disclosed that 84% of the individuals on hydralazine, alongside 62% of those on labetalol, needed only one dose to effectively control BP; however, the amount of time that each drug took to realize the targeted BP was not evaluated [65]. It's worth noting that only one of the mentioned studies was conducted in Nigeria [52].

Hydralazine and labetalol failure rates

In a study conducted by Deka [55], it was reported that the hydralazine group had a 4% failure rate, while the labetalol group had a 6% failure rate. However, this difference was not statistically significant. Mmom et al. [52] observed that 40% of patients placed in the labetalol group, along with 13.33% of those placed in the hydralazine group, required crossover therapy due to persistent hypertension, and the difference was found to be statistically significant. The author concluded that hydralazine was more effective than labetalol.

Numbur et al. [53], on the other hand, did not find a significant difference between the two drugs. They reported that 4.8% of patients assigned to the hydralazine group, as well as 3.2% of patients assigned to the labetalol group, required crossover therapy. Another study by Marbie et al. [62] compared hydralazine and labetalol in the management of acute hypertension during pregnancy. They enrolled 60 peripartum participants that had 110mmHg and higher diastolic BP, and they were randomized into a two-to-one ratio with the objective of receiving repeated administration of hydralazine (5mg) or labetalol (20-80mg) up to when 110mmHg diastolic BP or below was attained. The study disclosed a total of four treatment failures with regard to the labetalol group (N=40), even as none was reported in the hydralazine group (N=20) [54]. The researchers, therefore, arrived at the conclusion that labetalol was an effective and safer option than hydralazine in the treatment of hypertension during the peripartum period. Nonetheless, the researchers did not evaluate the number of dosages needed and the time taken to realize the targeted BP, and the randomization was in favor of labetalol (40:20) [54]. This new study aims to address these limitations by using balanced randomization (1:1) to eliminate bias. Garden et al. [63] found in their study that, in five out of six participants, labetalol led to a smoother, even, and gradual reduction in BP to the desired normal levels with insignificant side effects. Nonetheless, it was also found that in one out of three participants on hydralazine attained satisfactory BP control; however, for the last 4 participants, the discontinuation of treatment was recommended due to adverse effects such as maternal hypotension and fetal tachycardia. Although the randomization in this study was balanced, the sample size was too small to draw a conclusive result, and the use of continuous infusion of hydralazine, which may affect the bioavailability, onset of action and half-life of the drugs, differed from the methodology of this new study.

Adverse effects of hydralazine and labetalol

A meta-analysis [11, 14] comparing hydralazine with other antihypertensive drugs for severe gestational hypertension revealed that hydralazine was linked to a higher incidence of maternal hypotension, cesarean section, abruption placenta, maternal oliguria, and poor APGARscore. However, the study conducted by Magee et al. [45] concluded that although the results were not strong enough to provide definitive guidance for clinical practice, they did not support the use of hydralazine as the first-line antihypertensive agent for severe hypertension.

Contrarily, a recent Cochrane analysis [18], which only included studies with lower potential for bias and focused on women with severe hypertension in pregnancy, did not support the previous conclusion. This suggests the need for further research, leading to the motivation behind our study to provide additional evidence either supporting or refuting the use of these drugs. Deka et al. reported the absence of maternal hypotension with both agents [18, 54-55]. They also noted that patients who experienced a fresh stillbirth in either the hydralazine or labetalol group were individuals who were being treated for abruption placenta before the administration of either drug. As a result, they concluded that there was no superiority of one agent over the other [55-57].

Vigil De Gracia et al. [45], Numbur et al. [18], and Khan et al. [56], on the other hand, reported a higher frequency of headache, palpitations, and maternal tachycardia with the use of hydralazine in comparison to labetalol. Nevertheless, no statistically significant difference was reported in relation to the frequency of nausea and vomiting. It is worth noting that they did not evaluate dizziness and poor APGAR score as measures of drug safety. Comparable adverse maternal side effects resulting from the use of these medications have been reported in similar studies [52, 64-70]. In Nigeria, Mmom et al. [52] conducted a study to evaluate the effectiveness of IV hydralazine and IV labetalol in reducing and controlling acute hypertension in pregnancy. They found that IV hydralazine acted faster compared to IV labetalol with regard to reduction of BP in pregnancies marked with acute hypertension, devoid of any increment in the adverse effects. The researchers recommended hydralazine as the first-line medication for managing acute hypertension in pregnancy in Port Harcourt, Nigeria. However, it should be noted that the overall dosage of hydralazine administered surpassed the maximum daily dose of 40mg per day (10mg five times), which may account for the three early neonatal deaths reported in the study. Additionally, the sample size was small. Dizziness was not reported in the labetalol group, unlike the hydralazine group, where three patients reported dizziness.

Although criticized for their small sample size, Khan, Hafeez, and Farah [56] observed in 2017 that the maternal hypotension incidence, irregular fetal heart patterns, nausea, and vomiting were similar for both drugs. However, headache and maternal tachycardia were more prevalent in the hydralazine group. The sample size was also a limiting factor in the study. Additionally, Shabnum et al. [61] disclosed that 77.8% of women on hydralazine and 22.2% on labetalol experienced maternal hypotension and dizziness, which was statistically significant. However, the study was not adequately powered to account for fast and slow acetylators of hydralazine, which could influence the drug's bioavailability and side effects. The authors concluded that until adequate evidence is available, the antihypertensive agents' choice for acute hypertensive emergencies in pregnancy should be based on what has been acknowledged with regard to the adverse side effects of the medications and familiarity with a particular agent [55]. Our findings in this new study have added to the existing knowledge on the subject.

Study justification

Hypertensive disorder during pregnancy is considered a key cause of maternal mortality and morbidity at Central Hospital Benin City [17]. Globally, approximately half of these cases are attributed to eclampsia and preeclampsia. The incidence of eclampsia at Central Hospital Benin City is 3% of deliveries [17], which is higher compared to other healthcare facilities in Nigeria. For instance, some authors reported a rate of 1.2% of deliveries [68], Onuh at the University of Benin Teaching Hospital reported a 1.32% incidence rate [69], and Efetie lately reported a 0.82% incidence rate [70]. Sustained elevation in BP poses a significant risk of developing preeclampsia and eclampsia during pregnancy. To prevent these complications, fast-acting antihypertensive medications are administered.

At our center, IV hydralazine was the favored drug for managing acute hypertension during pregnancy until the introduction of IV labetalol in 2011. However, the cost of a 300mg dose of labetalol is approximately 35 times higher than a 40mg dose of hydralazine. Central Hospital Benin switched from IV hydralazine to IV labetalol without considering its cost-effectiveness in this context. Labetalol is expensive, and in Sub-Saharan Africa, many women require affordable and effective antihypertensive drugs. This is because over 70% of healthcare expenses in this region are covered through out-of-pocket payments [67]. As a result, patients who cannot afford labetalol often choose to defer hospital admission for stabilization after receiving adequate counseling, opting to stay at home. Unfortunately, this delay in seeking medical care often leads to complications such as eclampsia and cerebrovascular accidents associated with hypertension [71-73].

This study is necessary due to the decision to switch from IV hydralazine to IV labetalol at our hospital without any evidence-based rationale. Consequently, the hospital's pharmacy department reduced the procurement of IV hydralazine and gradually shifted to stocking only IV labetalol. To determine the most effective drug with limited side effects on the study population between the two drugs, we conducted a randomized clinical trial that compared IV hydralazine to labetalol in relation to the management of acute hypertension in pregnancy.

Aims and objective

The aim of the study was to compare the efficiency of IV labetalol and hydralazine with regard to the management of acute gestational hypertension in pregnant women at a tertiary healthcare facility in Sub-Saharan Africa. The specific objectives of the research are as follows: 1) to determine the time required for IV hydralazine and IV labetalol to achieve a 150/100mmHg BP or below; 2) to determine the number of doses of labetalol and hydralazine needed to achieve a BP level of less than or equal to 150/100mmHg; 3) to record any adverse effects experienced by the mother; and 4) to document any adverse effects on the fetus.

The research hypothesis is as follows: null hypothesis - there was no statistically significant difference in BP control for women with severe gestational hypertension when comparing IV hydralazine and IV labetalol. Alternate hypothesis - there is a significant difference in the BP control in acute gestational hypertension patients when comparing IV hydralazine and IV labetalol.

## Materials and methods

Study design and setting

This study employed a prospective open-label randomized clinical trial to investigate acute hypertension control in pregnant women admitted at Central Hospital Benin, Benin City, Nigeria. The research was conducted in the labor and maternity wards of Central Hospital and Stella Obasanjo Hospital in Benin City, Edo State. These hospitals are tertiary care centers and serve as referral facilities for several states, including Ondo, Edo, Kogi, and Delta. The Central Hospital has a total of 60 obstetric beds and 42 gynecological beds and has an average delivery rate of 4,000 infants per year. Consequently, Stella Obasanjo Hospital is equipped with 22 gynecological beds and 32 obstetric beds and has an average delivery rate of 3,500 infants per year. The prevalence of gestational hypertension in this center is 9% [17]. According to local management guidelines, pregnant women with severe gestational hypertension are usually admitted to the maternity ward, where investigations are conducted to rule out pre-eclampsia. Intravenous labetalol is used to lower blood pressure, starting with a slow administration of 25mg over five minutes. Subsequent doses of 50mg, 75mg, 75mg, and 75mg are given until the blood pressure is ≤150/100mmHg. Bp measurements were repeated every 20 minutes after the initiation of intravenous labetalol. Women with a gestational age less than 34 weeks are conservatively managed with steroid treatment, oral antihypertensives, and intermittent fetal kick chart monitoring as long as their blood pressure remains consistently ≤150/100mmHg for 24 hours without maternal or fetal complications. In cases where desired BP was not achieved, intravenous hydralazine (therapy switching) is administered. For women with a gestational age greater than 34 weeks and persistent hypertension, induction of labor was performed if the cervix was favorable, and emergency cesarean section was conducted when indicated per local guidelines. Maternal pulse rate and fetal heart rate are monitored using a Doppler ultrasound every 15 minutes.

The trial was conducted from October 2018 to March 2019 in the labor and maternity wards of the Department of Obstetrics and Gynecology unit at Central Hospital and Stella Obasanjo Hospital in Benin City. The study duration involved 247 pregnant women with gestational hypertension who were admitted to the labor and maternity ward for conservative management, stabilization, and delivery. Of the 247 women, 120 women satisfied the study inclusion criteria leading to their enrolment in the study, averaging approximately 24 women per month, six women per week, and one to two women per day in both centers.

Inclusion Criteria

The study included consenting adults aged 18-45 years with ≥28 weeks gestational age. The gestational age of ≥28 weeks was chosen given that pre-eclampsia mainly occurs during the second half of pregnancy (characteristically after 27 weeks of pregnancy). The fetus had to have a normal heart rhythm of 110 to 160mmHg, along with the participant having a normal maternal heart rate of >60 bpm to <120 bpm. Given that this study sought to compare the efficiency of IV hydralazine to IV labetalol, the participants were placed in one class of antihypertensives, either hydralazine or labetalol. Additional antihypertensives were not evaluated, and no women on them were included in the study.

Exclusion Criteria

Participants with recognized and diagnosed drug allergies, asthma, extant heart block and congestive cardiac failure history, maternal co-morbidities, including renal failure, chronic hypertension, diabetes, and hemoglobinopathy, as well as acute pre-eclampsia, fetal distress, eclampsia, and various abnormalities disclosed during laboratory assessment were excluded.

Sample Size

For the present study, the sample size was determined through the use of comparative study formula. The equation used was N = 2.Z^2.P.Q / d^2, where N represents the desired minimum sample size per group.

In the formula, Z corresponds to a confidence level of 95% and is taken as 1.96. P represents the prevalence of severe gestational hypertension in Nigeria, which was reported as 1.9% in a previous study. Q is computed as 100 - P. The desired accuracy level is set at 5%, and indicated by d. That is, the calculation is presented as follows: N = (2 * (1.96^2) * (1.9/100) * (98.1/100) / (0.05^2)). Simplifying further: N = 2 * 3.841 * 0.018 * 0.981 / 0.0025. This leads to N = 1431.848 / 25, resulting in N = 57.27. Rounded up, the minimum sample size per group is 57. Considering a 5% attrition rate, 60 subjects were recruited for each group, totaling to 120 study participants.

The study included women admitted to the labor and maternity ward with severe pregnancy-induced hypertension. Eligible participants underwent a detailed medical history assessment, physical examination (including general and systemic examination), and various investigations such as a full blood count, peripheral blood film, liver function test, serum electrolyte analysis, urea and creatinine measurement, urine dipsticks for significant proteinuria, and serum uric acid assessment. These measures were taken to select suitable participants for the study.

After counseling, eligible participants provided written informed consent and were randomly allocated to either of the two groups: the intravenous hydralazine group (group X) or the intravenous labetalol group (group Y). Randomization was performed using computer-generated numbers assigned to each participant. The sequential placing of the numbers in opaque envelopes by the researcher, and the subsequent sealing and requesting of the participants to select one enabled unbiased allocation of the participants to the groups. This process continued until all participants were allocated to a group. Following group assignment, intravenous access was established using a green cannula (18G), and the appropriate medication was administered to each group.

For group X (intravenous hydralazine group), a slow bolus of 5mg of intravenous hydralazine was administered over a period of five minutes. To prepare the dosage, 0.5ml of hydralazine was withdrawn using a 5ml syringe and diluted with 4.5ml of sterile water. The diluted solution was then administered at a rate of 1ml per minute. In instances where the preferred blood pressure levels were not realized within 20 minutes following the administration of the first dose, a second dose of 5mg of intravenous hydralazine was administered slowly over five minutes. If the target blood pressure was still not attained after the second dose, a maximum of five doses (5mg each) were given slowly over five minutes. If the desired blood pressure was not reached after this point, a combination of antihypertensive drugs (crossover therapy) was employed to control blood pressure. The specific brand of hydralazine used was Mack-hydralazine hydrochloride, which contains 20mg of hydralazine in a 2ml vial manufactured in New Delhi, India. The drugs were purchased from the Central Hospital Benin pharmacy in one batch to ensure continuous availability throughout the study.

Within the Y group, the administration of intravenous labetalol was performed as follows: a slow 25mg bolus dose of labetalol administration over five minutes (1ml per minute), followed by the administration of 50mg (2ml per minute) in instances where no efficiency is reported in 20 minutes. If necessary, an additional dose of 75mg (3ml/minute) was given every 20 minutes, with 300mg being the maximum. In case the target blood pressure level (BP ≤150/100mmHg) was not realized, a mixture of antihypertensive agents was administered. Blood pressure measurements were taken every 10 minutes, starting from the time of drug administration, and continued for up to two hours after the last dose or until the target blood pressure was reached. The time of drug initiation and the duration it took to achieve the desired BP were recorded using a stopwatch.

The specific brand of labetalol used was called Labet, which is manufactured in Bangladesh and comes in a 10ml container containing 50mg of labetalol. The drug was obtained from the hospital pharmacy in a similar manner as hydralazine. Throughout the entire process, all patients were actively monitored, with Bp measurements taken every 10 minutes and fetal heart rate measurements taken every 15 minutes for up to two hours after the last dose. The general condition of the participants was also taken into consideration.

The monitoring of the intermittent fetal heart rate occurred during the treatment using a handheld Doppler device. If an abnormal fetal heart rate/rhythm or a compromised maternal condition was detected, the trial protocol was discontinued, and appropriate standard interventions were carried out to ensure the safety of the mother and baby. Participants with uncontrollable blood pressure or near-term gestational age underwent labor induction, even as participants with aptly controlled blood pressure levels at a distance from term were placed under observation in the maternity ward, receiving oral antihypertensive medications, corticosteroids, and fetal kick charts. At the end of the study protocol, the participants were required to complete the provided questionnaire on the potential side effects we may have missed that they experienced during the trial period.

Outcomes

The principal outcome evaluated in the present study concentrated on aspects that included the duration required for intravenous administration of labetalol and hydralazine to lower BP to 150/100mmHg or below, the number of doses essential to realizing the targeted blood pressure level, and the failure rate of both drugs. The secondary outcome measures centered on evaluating the adverse effects of the drugs on the mothers, fetuses, and newborns delivered during the study period.

Data collection

To ensure accurate data collection, a structured data collection form was utilized to document all measurements and biodata of the participating individuals. Section A involved recording their biodata and socio-demographic parameters, including BP at enrollment and drug history. Section B contained information on the timing, the dosage of the trial drugs administered, as well as the corresponding blood pressure readings at the time of administration. Section C documented the number of doses required, failure rate, APGAR score at one and five minutes, adverse effects, mode of delivery, neonatal admission, and early neonatal death.

The research assistants involved in this study underwent a comprehensive training program lasting a minimum of two days (two hours per day). The training encompassed various aspects, such as obtaining consent, BP measurement techniques, questionnaire completion, reconstitution of drugs, and drug administration.

Prior to the initial BP measurement, women were granted a five-minute quiet rest. The initial BP was then measured with the individual in a seated position, ensuring that the cuff was placed at the level of the heart. Subsequent measurements of BP were recorded while the patient was lying in the left lateral position, even as diastolic and systolic BB were indicated using Korotkoff sounds I (first sound) and V (sound disappearance), correspondingly. In cases where a notable discrepancy existed between the fourth (muffling) and fifth (disappearance) Korotkoff sounds, with the fifth sound approaching zero, the fourth sound was regarded as indicative of diastolic BP. The BP cuff was deflated and left on the upper arm for subsequent BP measurements.

Data analysis

For this study, we conducted data analysis through the use of SPSS v. 23 statistical software (IBM Inc., Armonk, New York). The data analysis was carried out on the basis of the intent-to-treat concept. The data distribution normality was evaluated through the use of the Kolmogorov-Smirnov test. Further, the presentation of categorical variables was mainly in percentages and numbers, even as the expression of continuous was done in the form of standard deviation and mean. The non-normal data was mainly described using the median. Additionally, we utilized independent sample t-tests in comparing normally distributed data means alongside the Mann-Whitney U test that we utilized for the analysis of ordinal data. Fisher's exact and Chi-square tests were also employed in analyzing categorical variables. Each test was two-sided, and a p-value of <0.05 was regarded as statistically significant.

Ethical considerations

The study's approval was obtained from the Central Hospital Benin's ethical committee, approval number HMB10092018. We adhered to the various ethical considerations in this study, including the general ethical principles that are applicable to human study subjects [68]. The patients were only enrolled to participate in the research only after getting sufficient information and offering written informed consent. The participants were neither forced nor persuaded to partake in the study, even as their individual rights to withdraw or participate in the study were wholly respected. Patients were not required to bear any costs related to purchasing drugs. Universal safety precautions were observed throughout the study. Medications beneficial for blood pressure management in cases of persistently high pressure were not withheld, and patients' management was not impacted by their refusal to partake in the research.

## Results

The uniformity of the study population for the two groups is reflected by the baseline characteristics presented in Table 1. The participants in both groups demonstrated similarities in terms of age, parity, gestational age, and booking status. Moreover, the baseline pretreatment measurements of Bp, pulse rate, and fetal heart rate were found to be comparable between groups, with no observed significant differences.

**Table 1:** General characteristics of the study populations Significant p-value ≤ 0.05; BPM = beats per minute; DBP = diastolic blood pressure; SBP = systolic blood pressure; SD = standard deviation; antenatal booking status = antenatal enrollment status; FHR = fetal heart rate

Characteristics	Hydralazine group		Labetalol group		p-value
	Total (n +/- SD)	%	Total (n +/- SD)	%	
Age (years) mean	25.57 +/- 5.369		25.750 +/- 5.170		0.849
< 25	37	61.70%	34	56.70%	
>25	23	38.30%	26	43.30%	0.577
Parity	0.82 +/- 0.965		0.68 +/- 1.00		0.459
Nulliparous	34	56.70%	36	60.00%	
Primiparous	9	15.00%	13	21.70%	
Multiparous	17	28.30%	11	18.30%	0.355
Gestational age (weeks)	33.97 +/- 3.844 weeks		34.78 +/- 3.88 weeks		0.249
<37 weeks	41	68.30%	31	51.70%	
>37 weeks	19	31.70%	29	48.30%	0.062
Antenatal booking status (Antenatal enrollment status)					
Unbooked	15	25.00%	12	20.00%	
Booked	45	75.00%	48	80.00%	0.512
SBP at enrollment	170.67 +/- 9.719mmHg		171.67 +/- 13.80 mmHg		0.647
DBP at enrollment	112.67 +/- 6.342mmHg		113.67 +/- 8.82mmHg		0.477
Pulse rate at enrollment	84.27 +/- 9.303/minutes		82.57 +/- 6.946/minutes		0.25
FHR at enrollment	142.000 +/- 6.587 BPM		139.87 +/- 6.419 BPM		0.075

Table 2 presents the changes in mean arterial BP. The pretreatment mean arterial BP was 131.717±10.371 mmHg in the hydralazine group and 133.45±8.730 mmHg in the labetalol group (p=0.324). Following treatment, the mean arterial BP was 111.050±17.072 mmHg in the hydralazine group and 115.583±14.769 mmHg in the labetalol group (p=0.122). The mean arterial BP changes were 20.136±12.881 mmHg in the hydralazine group and 18.050±14.038 mmHg in the labetalol group (p=0.4000).

**Table 2:** Mean arterial blood pressure changes after drug therapy Significant p-value ≤ 0.05, BP = blood pressure

Group	Mean arterial BP changes (mmHg)		
	Before	After	Mean change
Hydralazine group (µ ± SD)	131.717±10.371	111.050±17.072	20.136±12.881
Labetalol group (µ ± SD)	133.45±8.730	115.583±14.769	18.050±14.038
p-value	0.324	0.122	0.4

Table 3 provides a comparison of the time required to normalize BP, the number of doses needed, and the failure rate. In the intravenous hydralazine group, it took approximately 45.80 ± 25.17 minutes to reach the target BP of ≤ 150/100 mmHg, whereas patients in the labetalol group required about 72.67 ± 41.80 minutes (P = 0.0001) (See figure 1 below).

**Table 3:** Time and number of doses to normalize BP Significant p-value, ≤ 0.05, SD= standard deviation, HTN= Hypertension

Outcome	Hydralazine group (minutes)	Labetalol group (minutes)	p-value	
Time to reach target blood pressure. (mean±SD)	45.80 +/- 25.17	72.67+/- 41.80	0.0001	
Number of doses required. (mean±SD)	1.72 +/- 0.904	3.72 +/- 1.782	0.0001	
Comparison of dosing frequency				
Single dose n(%)	27(45.0%)	14(23.3%)	0.02	
2 to 3doses n(%)	24(40.0%)	11(18.3%)	0.000439	
4 to 5doses n(%)	4(6.7%)	20(33.3%)	0.00003	
Persistent HTN n(%)	5(8.3%)	15(25.0%)	0.0257	
Total	60(100%)	60(100%)		

Intravenous hydralazine also demonstrated a lower requirement for doses (1.72 ± 0.904) compared to intravenous labetalol (3.72 ± 1.782) (p=0.008). Furthermore, persistent hypertension, despite receiving the maximum dose of the assigned medication, was observed in 8.3% of patients within the hydralazine group and 25% in the labetalol group (p=0.0143). In the hydralazine subset, 27 patients (45%) achieved BP control with a single dose, whereas in the labetalol group, this was the case for 14 patients (23.3%) (p=0.02). Additionally, 24 subjects (40%) in the hydralazine group and 11 (18.3%) in the labetalol group required two to three doses of their respective drugs to achieve BP control (p=0.0004). Moreover, four subjects (6.7%) in the hydralazine group and 20 (33.3%) in the labetalol group needed four to five doses of their respective drugs to reach the target BP (p=0.00003). In total, five patients in the hydralazine group and 15 in the labetalol subset experienced persistent hypertension despite receiving five doses of the assigned antihypertensive agent, resulting in a failure rate of 8.3% and 25%, respectively.

Table 4 presents the mode of delivery for the participants who underwent delivery. In the hydralazine group, 16 patients had a vaginal delivery, while nine required an emergency cesarean section. Conversely, in the intravenous labetalol group, 15 patients had a vaginal delivery, and 19 patients underwent an emergency cesarean section. The remaining patients, comprising 35 in the hydralazine group and 26 in the labetalol group, continued with conservative management as their blood pressure was under control and they were distant from their expected delivery date.

**Table 4:** Mode of delivery for the participants that were delivered Significant p-value ≤ 0.05, EMCS = emergency cesarean section

Characteristics	Hydralazine group, n	%	Labetalol group, n	%
Vaginal delivery	16	26.7%	15	25.0%
EMCS	9	15.0%	19	31.7%
Conservative management	35	58.3%	26	43.3%
Total	60	100%	60	100%
Chi-squared	2.284			
p-value	0.131			

Table 5 presents the indications for emergency cesarean section (EMCS). In the hydralazine group, four patients required EMCS due to persistent fetal tachycardia, while two subjects in the labetalol group had the same indication. Additionally, two patients in the hydralazine group and 13 in the labetalol group underwent EMCS due to failed induction resulting from persistent hypertension with an unfavorable cervix. Furthermore, cesarean section was done for three patients in the hydralazine group and four in the labetalol group due to slow progress in labor.

**Table 5:** Indication for EMCS Significant p-value ≤ 0.05, HTN = Hypertension

Indications	Hydralazine group	Labetalol group	p-value
Persistent fetal tachycardia.	4	2	0.943
Persistent HTN + unfavourable cervix (failed induction)	2	13	0.022
Slow labour progress	3	4	0.6465
Total	9	19	

Table 6 presents the adverse maternal outcomes observed in the two arms. None of the cases involved abruptio placentae. In the hydralazine group, four patients and two in the labetalol arm experienced hypotension, defined as systolic BP <90 mmHg or diastolic BP <60 mmHg, or both. Dizziness was reported by nine patients in the hydralazine group and two patients in the labetalol group. Headaches were complained of by 18 patients in the hydralazine group and eight in the labetalol group after medication administration. Additionally, 10 women in the hydralazine group and two in the labetalol group experienced nausea and vomiting. Notably, maternal tachycardia was observed in five subjects from the hydralazine arm, while none was observed in the labetalol arm. The occurrence of dizziness, nausea/vomiting, and headache was significantly higher in patients receiving hydralazine compared to those given labetalol. These differences were statistically significant (p=0.028, p=0.015, and p=0.028, respectively). No maternal deaths were reported among the women included in the study.

**Table 6:** Adverse maternal outcomes Significant p-value ≤ 0.05

Adverse effect	Hydralazine group	%	Labetalol group	%	p-value
Hypotension	4	6.7	2	3.3	0.402
Dizziness	9	15	2	3.3	0.0268
Nausea/ vomiting	10	16.7	2	3.3	0.015
Headache	18	30	8	13.3	0.028
Abruption placenta	0	0	0	0	
Maternal tachycardia	5	8.33	0	0	0.058

Table 7 illustrates the fetal outcomes. Among the babies in the hydralazine group, five had a first-minute APGAR score of less than seven, while two babies in the labetalol arm had the same score. Similarly, two babies in the hydralazine arm and one in the labetalol had a fifth-minute APGAR score of less than seven (p=0.855). Additionally, two neonates in the hydralazine group and one neonate in the labetalol group required admission to the special care baby unit (SCBU) due to birth asphyxia. Fortunately, no cases of perinatal mortality were recorded.

**Table 7:** Adverse fetal and neonatal outcomes Significant p-value ≤ 0.05, APGAR = appearance, pulse, grimace, activity, and respiration, NICU = neonatal intensive care unit

Fetal outcome	Hydralazine group	%	Labetalol group	%	p-value
1 -inute APGAR score <7	5	20.00%	2	5.9%%	0.1222
5 -inute APGAR score <7	2	8.00%	1	2.90%	0.5686
NICU admission	2	8.0%%	1	2.90%	0.5686

## Discussion

According to this study, intravenous hydralazine demonstrated faster efficacy than intravenous labetalol in treating acute onset severe hypertension during pregnancy. It requires fewer doses to reach the desired BP level, although it does come with more adverse effects compared to intravenous labetalol. The time taken to achieve the target BP in this clinical trial aligns with previous studies conducted in Port Harcourt by Mmom et al. [52], in Pakistan by Sabir and colleagues [59], and older research conducted by Magee et al. [14].

It is widely accepted that pregnant women with severe pregnancy-induced hypertension should receive antihypertensive medication promptly, within 40-60 minutes of diagnosis, to minimize the risk of cerebrovascular accidents [44, 61]. In this study, it took an average of 73 minutes for the labetalol group and 46 minutes for the hydralazine group to achieve the target blood pressure. The prolonged average time it took intravenous labetalol to reach the target blood pressure contradicts the objective of rapidly reducing blood pressure in hypertensive emergencies. These findings support the alternative hypothesis proposed in this study, which demonstrates the superiority of one drug over the other in achieving rapid blood pressure control. The results from this study align with the work conducted by Mmom et al. in Port Harcourt, Nigeria, who reported a mean time of 28 minutes and 75 minutes for intravenous hydralazine and intravenous labetalol, respectively, to normalize blood pressure. The longer time of 46 minutes, compared to 28 minutes in the present study, needed to achieve the target blood pressure might be attributed to the fixed dose of hydralazine (5mg) used in our trial. Previous studies that examined the efficacy of hydralazine and labetalol as antihypertensive agents generally favored the null hypothesis, indicating no significant superiority of one drug over the other in reducing blood pressure [45, 53-58]. Disparities in racial backgrounds and the influence of drug potency in tropical regions might explain this discrepancy. In a study conducted by Deka and colleagues [55], the mean time to achieve the target blood pressure was 32 minutes for intravenous hydralazine and 31 minutes for intravenous labetalol. The shorter time required to reach the target blood pressure in their study, compared to the present study, can be attributed to the more frequent dosing interval of 15 minutes, as opposed to 20 minutes in our study.

The effectiveness of antihypertensive medication can also be evaluated based on the dosage required to normalize blood pressure [1-3, 23-31, 45-59]. In the current study, it was found that an average of two doses of hydralazine and four doses of labetalol were necessary to achieve the target blood pressure. Similar results were reported by Mmom et al. [52] in Port Harcourt, Nigeria, where it took three doses of labetalol compared to a single dose of hydralazine to control blood pressure. However, these findings contradict earlier studies conducted by Ashe et al., Delgado and Vigil-De Gracia et al., which demonstrated no significant statistical differences in the number of doses required for both agents to achieve the target blood pressure [54,58]. The lower number of hydralazine doses needed to attain the target blood pressure in the Port Harcourt study compared to the current study may be attributed to the higher intravenous hydralazine dosage (10mg) used in their study, as opposed to 5mg used in the present study. In the present study, it was observed that 27 patients (45%) in the hydralazine group and 14 patients (23.3%) in the labetalol group achieved the target blood pressure after a single dose, which was a statistically significant difference (p=0.02). Additionally, 40% of patients in the hydralazine group and 18.3% in the labetalol group required two to three doses of the assigned medications to achieve the target blood pressure (p=0.000049). Furthermore, 6.7% of women in the hydralazine group and 33.3% in the labetalol group needed four to five doses to control their blood pressure. These findings are consistent with studies conducted by Khan et al.[59], Pooja et al. [60], and Shabnum et al. [61], which also demonstrated similar trends [4]. However, they differ from previous studies conducted by Ashe et al. [45], Nombur et al. [53], Delgado de Pasquale et al. [54], Deka et al. [55], and Vigil-de Gracia et al. [45], which reported no significant statistical differences in the number of patients requiring a single dose of the assigned medication to achieve the target blood pressure. 

In this study, the failure rate of treatment was 8.3% (five cases) in the hydralazine group and 25% (15 cases) in the labetalol group (p=0.0143). This finding aligns with a study conducted in Port Harcourt, Nigeria, by Mmom et al., where 40% of patients in the labetalol group and 13.33% in the hydralazine group required crossover therapy due to persistent hypertension [52]. However, previous research has shown no significant difference in failure rates and the need for crossover therapy between the two drugs [45, 53-55, 58]. 

The difference in results between this study and previous research could be attributed to variations in drug potency and racial differences in the study population. Labetalol has been found to be more effective in controlling hypertension among White individuals compared to Black individuals [50, 51, 52]. Notably, there were no significant differences observed in mean arterial blood pressure before and after administering medications, and the mean arterial change in blood pressure was also not statistically significant. Similar findings were reported in studies by Trivedi et al. and Delgado de Pasquale et al. [54, 64]. 

In this study, the rate of cesarean section was 19.9% higher in the labetalol group compared to the hydralazine group. This is not surprising, as failure to achieve blood pressure control often leads to emergency cesarean section, which is considered the fastest and safest delivery method in the study environment. Additionally, vaginal delivery was more common in the hydralazine group, as those who achieved the target blood pressure during labor were allowed to have a vaginal birth. These findings are consistent with previous studies [52], although other studies did not specifically evaluate the mode of delivery. 

The primary goal of treating acute, severe hypertension is to gradually reduce blood pressure to a safe level for both the mother and fetus while avoiding a rapid drop in blood pressure that can cause complications. In this study, maternal hypotension, dizziness, nausea, vomiting, and headache were significantly higher in the hydralazine group compared to the labetalol group. However, there were no cases of abruptio placenta. These findings align with documented side effects of hydralazine in previous studies [18, 53, 55, 66]. Maternal tachycardia was also significantly higher in the hydralazine group, whereas no cases of maternal tachycardia were observed in the labetalol group. This observation is consistent with the general belief that labetalol, as a non-selective beta-adrenergic blocking agent, leads to a dose-related decrease in blood pressure without reflex tachycardia and a significant reduction in heart rate [44]. These findings are in agreement with earlier studies and a Cochrane review on the efficacy of both drugs in hypertensive crisis during pregnancy [11, 14, 45]. However, some studies reported the absence of maternal hypotension with both agents [18, 54, 55]. The side effects observed in this study (dizziness, nausea, vomiting, headache, and maternal tachycardia) were not severe enough to require discontinuation of hydralazine between administrations. 

There was no statistically significant difference in the first- and fifth-minute AGPAR scores between the hydralazine and labetalol groups. Two newborns from the hydralazine group and one newborn from the labetalol group were admitted to the special care baby unit due to birth asphyxia. It is noteworthy that there were no perinatal deaths reported in either group, which aligns with previous studies [18, 54, 55]. Deka et al., Delgado De Pasquale et al., and Agida et al. found that patients who experienced a fresh stillbirth in the hydralazine or labetalol group had previously received management for abruption placenta before the administration of either medication [18, 54-55]. 

Based on the findings of this research, it was determined that the current price for a 40mg dose of hydralazine at the local hospital pharmacy is N200.00, equivalent to approximately $0.48 USD for two doses. In contrast, the price for a 300mg dose of labetalol is N7000.00, approximately $17 USD for four doses. These figures indicate a significant increase of approximately 94% in the cost of medication.

Prior to the year 2003, intravenous hydralazine was commonly prescribed as the initial treatment option [42-45, 66]. However, Magee and colleagues conducted a meta-analysis during that time and introduced intravenous labetalol as a viable alternative with a more favorable adverse effect profile [11, 14]. They also noted that the side effects of hydralazine resemble those of impending eclampsia [18]. Nevertheless, similar adverse effects have been observed with the use of labetalol in this and other studies [18, 37, 66]. Considering these comparable adverse effects, Duley et al., in a Cochrane review, concluded that neither drug demonstrated superiority over the other. Consequently, they advised clinicians to individualize their approach when prescribing antihypertensive medications for severe pregnancy-induced hypertension [18, 52].

Strengths, challenges, and limitations

The initial characteristics of the participants in both groups were similar. Despite a number of previous studies [1-20] suggesting a higher occurrence of severe gestational hypertension in women of advanced maternal age (>35 years), in our study, more than 59% of women with severe gestational pregnancy were below 25 years of age. This observation may necessitate additional research on why more younger women are developing severe gestational hypertension in comparison to older women. Though a number of previous studies had reported that un-booked women who had not received early diagnosis and antenatal supervision were highly prone to develop severe gestational hypertension [4, 33, 34], this study has disclosed that over 77% of women in the two groups had sought prenatal care in time and still developed gestational hypertension, even as most of the women recruited in this study resided in urban areas and had access to early prenatal care.

During the study, patients were closely monitored for a duration of two hours following the initial control of blood pressure or administration of the maximum dose. Extending the patient follow-up to 24 hours could potentially identify more cases of rebound hypertension and maternal adverse effects. Unfortunately, we did not evaluate urinary output in this study. It is worth noting that previous research has indicated a potential association between hydralazine usage and reduced renal blood flow.

Throughout the trial period, continuous electronic fetal monitoring was not employed for all the participants, which may have limited our ability to identify more adverse outcomes in newborns. Furthermore, it's important to acknowledge that the study was not conducted in a blind manner, leaving room for observer bias. We were unable to exclude fast acetylators who could have rapidly metabolized hydralazine, leading to reduced drug bioavailability. However, it is crucial to mention that individuals with a history of allergy to any of the drugs were excluded from the study.

Furthermore, the research study posed significant challenges in terms of drug procurement, patient recruitment involving travel between Central Hospital and Stella Obasanjo Hospital, and the need to await results of electrolytes, urea, and creatinine (E/U/Cr) test and liver function tests (LFT) before selecting patients for inclusion. In some cases, patients had already been started on antihypertensive medication before arriving at the labor ward without undergoing the necessary investigations to determine their eligibility for participation in the study. Such patients, upon arrival, who met eligibility criteria were included following the principal investigator's approval.

Recommendations

Although the study findings have indicated that IV hydralazine was effective in attaining the target BP in a shorter time and with fewer dosages compared to IV labetalol, IV hydralazine presented more adverse maternal side effects than IV labetalol. These findings highlight the need for careful consideration when selecting the appropriate medication. Considering the rapid control of blood pressure in women with severe gestational hypertension, who do not have allergies to intravenous hydralazine, it may be advisable to consider hydralazine as the preferred initial medication. Nevertheless, it is essential to conduct further research to explore alternative options to intravenous hydralazine. This should involve large-scale community-based studies with adequate statistical power, encompassing a wider age range of participants. In addition, conducting multi-centered double-blind trials will enhance the search for a suitable alternative. To ensure robust results, future trials should implement stratified randomization based on individual blood pressure values. It is crucial to analyze subgroups of women with chronic hypertension, non-proteinuric hypertension, and pre-eclampsia separately. Moreover, women who achieve blood pressure control through intravenous hydralazine should be transitioned to oral hydralazine and monitored until delivery, while those who achieve blood pressure control through intravenous labetalol should be transitioned to oral labetalol for comparative purposes.

## Conclusions

This study, therefore, concludes that intravenous hydralazine acts faster than intravenous labetalol and requires fewer doses of drugs than intravenous labetalol for acute control of blood pressure in women with severe gestational hypertension. However, intravenous hydralazine was associated with more maternal side effects such as hypotension, dizziness, nausea, vomiting, and headache compared to intravenous labetalol, but no final dose reduction or drug discontinuation was required due to major side effects. Furthermore, intravenous hydralazine has no significant adverse effect on the baby.

## Appendices

Data collection form

**Table 8:** Section a: baseline characteristics of women in the study groups, including their gestational age, hypertension history and anti-hypertensives used before

Characteristics	
STUDY GROUP (X or Y)	
Maternal age	
Parity	
Gestational age	
Booking status	
Average SBP at enrollment	
Average DBP at enrollment	
Mean arterial BP at enrolment	
Mean arterial BP after enrolment	
Changes in mean arterial BP	
Fetal heart rate at enrollment.	
Pulse rate at enrollment.	
Fetal heart rate after enrollment	
Use of antihypertensive drugs before admission	YES NO
Use of corticosteroind	YES NO
Use of magnesium sulfate.	YES NO

	ENROLLMENT	1^ST^ DOSE	2^ND^ DOSE	3^RD^ DOSE	4^TH^ DOSE	5^TH^ DOSE,
TIME						
BP READING						

NUMBER OF DOSES REQUIRED.	
TIME TO REACH TARGET BP (MINUTES)	
PERSISTENT HYPERTENSION	YES NO
NEED FOR COMBINATIONS OF ANTIHYPERTENSIVES.	Yes NO
MATERNAL HYPOTENSION	YES NO
NAUSEA	YES NO
HEADACHE	YES NO
ABRUPTIO PLACENTA	YES NO
VAGINAL DELIVERY	YES NO
EMCS	YES NO
BIRTH WEIGHT	
1 MINUTE APGAR SCORE<7	YES NO
5 MINUTE APGAR SCORE<7	YES NO
NICU ADMISSION	YES NO
PERINATAL DEATH	YES NO

**Table 9:** Group Statistics, including maternal and gestational ages of the participants, average BP at enrolment and after enrolment, dosages administered, and time taken to realize the target BP

Group Statistics					
	Drug	N	Mean	Std. Deviation	Std. Error Mean
maternal age	Labetalol	60	25.48	5.044	.651
	Hydralazine	60	25.57	5.369	.687
parity	Labetalol	60	.63	.901	.116
	Hydralazine	60	.82	.965	.125
gestational age	Labetalol	60	34.78	3.880	.50242
	Hydralazine	60	33.97	3.844	.49281
average SBP at enrollment	Labetalol	60	171.67	13.80	1.79177
	Hydralazine	60	170.67	9.719	1.24173
average DBP at enrollment	Labetalol	60	113.8333	8.84742	1.14220
	Hydralazine	60	112.6667	6.34240	.81880
fetal heart rate at enrollment	Labetalol	60	139.9333	6.44104	.83153
	Hydralazine	60	142.1000	6.66308	.86020
pulse rate at enrollment	Labetalol	60	82.9333	7.27398	.93907
	Hydralazine	60	83.7667	8.97523	1.15870
total number of doses requred	Labetalol	45	3.22	1.782	.266
	Hydralazine	55	1.72	.904	.128
time to reach target BP	Labetalol	45	72.6667	41.80039	6.23123
	Hydralazine	55	45.8000	24.58554	3.47692
birthweight of babies delivered	Labetalol	34	2.9375	.52348	.09254
	Hydralazine	25	2.7125	.48425	.12106
fetal heart rate after enrollment	Labetalol	60	146.5833	8.54161	1.10272
	Hydralazine	60	147.2333	6.78075	.87539
mean arterial BP at enrollment	Labetalol	60	133.4500	8.73028	1.12707
	Hydralazine	60	131.7167	10.37123	1.33892
mean arterial BP after enrollment	Labetalol	60	115.5833	14.76860	1.90662
	Hydralazine	60	111.0500	17.07255	2.20406
mean arterial Bp changes after enrollment	Labetalol	60	18.0500	14.03799	1.81230
	Hydralazine	60	20.1356	12.88070	1.67692

**Table 10:** Statistical analysis Table indicating the findings of the Levene's test for equality of variances and t-tests for equality of means

		Levene’s Test for Equality of variances		t-test for Equality of means						
		F	Sig.	t	df	Sig(2-tailed)	Mean Difference	Std.Error Difference	95% CI of the differences	
									lower	upper
Age	Equal variances assumed	.158	.692	.191	118	.849	.183	.962	-1.722	2.089
	Equal variances not assumed			.191	117.8	.849	.183	.962	-1.722	2.089
Parity	Equal variances assumed	.638	.426	-743	118	.459	-133	.179	.489	.222
	Equal variances not assumed			-743	117.8	.459	-133	.179	.489	.222
Gestational age	Equal variances assumed	.026	.873	1.158	118	.249	.81667	.70513	.57968	2.21302
	Equal variances not assumed			1.158	118.0	.249	.81667	.70513	.57968	2.21302
Systolic BP at enrollment	Equal variances assumed	3.406	.067	.459	118	.647	1.0000	2.17891	-3.31483	5.31483
	Equal variances not assumed			.459	106.0	.647	1.0000	2.17891	-3.31483	5.31383
Diastolic BP at enrollment	Equal variances assumed	4.519	.036	.713	118	.477	1.0000	1.40285	-1.77802	3.77802
	Equal variances not assumed			.713	107.1	.477	1.00000	1.40285	-1.78095	3.78095
Fetal HR at enrollment	Equal variances assumed	.076	.784	-1.797	118	.075	-2.13333	1.18738	-4.48467	.21800
	Equal variances not assumed			-1.797	117.9	.075	-2.13333	1.18738	-4.48467	.21802
Pulse rate at enrollment	Equal variances assumed	5.673	.019	-1.156	118	.250	-1.700000	1.47085	-4.61269	1.21269
	Equal variances not assumed			-1.156	110.7	.250	-1.700000	1.47085	-4.61468	1.21468
Doses required to control BP	Equal variances assumed	19.546	.000	1.958	103	.053	.465	.238	.006	.937
	Equal variances not assumed			1.924	85.942	.058	.465	.242	.015	.946
Fetal heart rate after enrollment	Equal variances assumed	.188	.665	-333	118	.740	-46667	1.40199	-3.24299	2.30965
	Equal variances not assumed			-333	111.7	.740	-46667	1.40199	-3.24460	2.31127
Mean arterial BP at enrollment	Equal variances assumed	.625	.431	1.094	118	.324	1.91667	1.75138	-1.55155	5.38488
	Equal variances not assumed			1.094	114.9	.324	1.91667	1.75138	-1.55251	5.38585
Mean arterial BP after enrollment	Equal variances assumed	.089	.766	1.753	118	.122	5.00000	2.85271	-.64914	10.64914
	Equal variances not assumed			1.753	116.5	.122	5.00000	2.85271	-.64990	10.64990
Mean changes in arterial BP	Equal variances assumed	.649	422	-871	118	.400	-2.03333	2.33466	-6.65660	2.58993
	Equal variances not assumed			-871	116.8	.400	-2.03333	2.33466	6.65709	2.59042
